# Response and conflict expectations shape motor responses interactively

**DOI:** 10.1007/s00221-024-06920-w

**Published:** 2024-09-24

**Authors:** Annika E. Sauter, Adam Zabicki, Thomas Schüller, Juan Carlos Baldermann, Gereon R. Fink, Paola Mengotti, Simone Vossel

**Affiliations:** 1 Institute of Neuroscience & Medicine (INM-3), Cognitive Neuroscience, Forschungszentrum Jülich, Leo-Brandt-Str. 5, 52425 Jülich, Germany; 2 Department of Neurology, Faculty of Medicine and University Hospital Cologne, University of Cologne, 50937 Cologne, Germany; 3 Department of Psychiatry and Psychotherapy, Faculty of Medicine and University Hospital Cologne, University of Cologne, 50937 Cologne, Germany; 4 Department of Psychology, Faculty of Human Sciences, University of Cologne, 50923 Cologne, Germany; 5 Department of Psychiatry and Psychotherapy, Faculty of Medicine, Medical Center, University of Freiburg, 79104 Freiburg, Germany

**Keywords:** Motor cueing, Flanker interference, Cue validity, Congruency proportion, Motor reprogramming, Conflict resolution

## Abstract

Efficient responses in dynamic environments rely on a combination of readiness and flexibility, regulated by anticipatory and online response control mechanisms. The latter are required when a motor response needs to be reprogrammed or when flanker stimuli induce response conflict and they are crucially modulated by anticipatory signals such as response and conflict expectations. The mutual influence and interplay of these control processes remain to be elucidated. Our behavioral study employed a novel combined response cueing/conflict task designed to test for interactive effects of response reprogramming and conflict resolution and their modulation by expectations. To this end, valid and invalid response cues were combined with congruent and incongruent target flankers. Expectations were modulated by systematically manipulating the proportions of valid versus invalid cues and congruent versus incongruent flanker stimuli in different task blocks. Reaction time and accuracy were assessed in thirty-one healthy volunteers. The results revealed response reprogramming and conflict resolution interactions for both behavioral measures, modulated by response and conflict expectations. Accuracy decreased disproportionally when invalidly cued targets with incongruent flankers were least expected. These findings support coordinated and partially overlapping anticipatory and online response control mechanisms within motor-cognitive networks.

**Supplementary Information:**

The online version contains supplementary material available at 10.1007/s00221-024-06920-w.

## Introduction

Our motor responses result from competitive advantages over alternative options, constantly updated to optimize behavior. This process is guided by preparing relevant motor responses and prioritizing the optimal response among alternatives (Brown et al. 2011; Cisek and Kalaska 2010). An optimal response, however, is situational and subject to internal and external dynamics. A constant influx of information is concurrently transformed into viable response plans to accommodate these dynamics. Efficient responding involves balancing between readiness and flexibility based on current expectations. Readiness is promoted by anticipatory response control, allowing for adequate preparation. Complementarily, online control mechanisms enable flexibility during response execution, in case expectations are violated or distractions occur (Aron 2011; Brown et al. 2011; Cisek and Kalaska 2010).

The flexible online control can particularly be probed in two situations: First, in situations that require the suppression of the prepared response and the programming of an alternative (Mars et al. 2007). Second, in case of competing response representations that demand conflict resolution (Duque et al. 2013; Heekeren et al. 2008). Experimentally, response preparation and reprogramming have been assessed with response cueing tasks such as motor versions of the Posner attention task (Posner 1980; Rosenbaum & Kornblum, 1982; Rushworth et al. 1997; 2001). In these tasks, probabilistic response cues induce motor preparation of the indicated response. Valid cues lead to faster responses with fewer errors, whereas invalid cues result in slower and more erroneous responses. These behavioral (reaction time (RT) or accuracy) differences between invalid and valid trials are termed the “validity effect”. In the case of response conflict, similar response options compete for execution and require additional selection effort (Cisek and Kalaska 2010). This situation can be created in an experimental setting by introducing irrelevant distractor stimuli associated with alternative responses, as in the Erikson flanker task (Eriksen and Eriksen 1974). In the arrow version of this task, a central target arrow is “flanked” by peripheral distractor arrows with either congruent or incongruent directions. Behavioral differences between incongruent and congruent trial types are termed the “congruency effect”.

Beyond online control, response reprogramming and conflict resolution are affected by anticipatory control mechanisms through expectations. In the cueing task, the frequency of valid and invalid cues (i.e., the expectation that the cue will be valid in a given trial) modulates the validity effect through varying preparation strength and surprise, even when these probabilities are unknown and need to be inferred from trial-wise observations (Bestmann et al. 2008; Brown et al. 2011; Kuhns et al. 2017; Mengotti et al. 2020; Vossel et al. 2014). Bestmann and colleagues (2008) showed that RTs, as well as corticospinal excitability (CSE), covaried with information-theoretic measures of entropy (i.e., context-derived expectations) and surprise (i.e., how unexpected the event is within this context). Kuhns et al. (2017) observed that the size of the validity effect increases with an increasing probability of valid cues (i.e., when invalid trials are less expected). Similarly to the validity effect, expectations regarding conflict occurrence (i.e., the frequency of incongruent trials) influence the congruency effect, with increased error rates for infrequent incongruent trials (Derosiere et al. 2018; Duque et al. 2016; Logan 1980). The exact mechanisms underlying the proportion congruency effect are still debated (Schmidt 2013; Braem et al. 2019). Whereas it has initially been attributed to anticipatory conflict adaptation (Botvinick et al. 2001), alternative explanations involve modulations due to stimulus-response contingency or temporal learning. Still, conflict adaptation and lower-level learning processes may also go hand in hand (Braem et al. 2019), and it has been shown that the effect of varying congruency proportions can be described by formal volatility-based reinforcement learning schemes that predict control demands (Jiang et al. 2015), similar to the validity proportion effect in cueing tasks (Kuhns et al. 2017). It is currently unclear whether a common control mechanism coordinates online response reprogramming and conflict resolution and their modulation by expectations. Hence, by manipulating these factors within the same task, we wanted to test at which levels mutual influences (interactions) may occur. More generally, it has been proposed that dynamic response requirements align with a neural system that can operate in parallel processing streams (e.g., Cisek 2007; Cisek and Kalaska 2010). Rather than operating as a functionally designated and locally confined mechanism, response control may arise from the interactions among distributed subprocesses (Ridderinkhof et al., 2011).

In line with this view, a recent study found partially distinct but coordinated neural signatures of anticipatory and online control (Asanowicz et al. 2022). Hence, distributed neural subnetworks may encode the selection and specification of response plans (e.g., Crammond and Kalaska 2000). An integral control mechanism may involve mutual inhibition and excitement (Cisek 2006), as well as neural interconnections across functional boundaries (Haber 2003). Furthermore, it is well known that an intricate balance between inhibitory and disinhibitory forces in the basal ganglia loops shapes cortically-generated response programs. Cortical regions such as the anterior cingulate cortex (ACC) and fronto-parietal areas are robustly activated by interference tasks including the flanker task (Isherwood et al. 2021), and subcortico-cortical interplays may be relevant for coordinating response control subprocesses. In contrast to the effect of spatial attention cues on conflict processing, which has been investigated by numerous behavioral and neuroimaging studies using variants of the Attention Network Test (ANT; Fan et al. 2002), and for which interactive “bottleneck”-effects have been reported in the insula (Trautwein et al. 2016), studies on the interplay of response cues and flankers are scarce. Since pathological disturbances of these motor-cognitive subprocesses, e.g., in Parkinson’s Disease, generate maladjusted response outcomes (e.g., Aron et al. 2016; Bonnevie and Zaghloul 2019; Galea et al. 2012), a further understanding of the underlying control mechanisms and subprocesses is also relevant from a clinical perspective.

Previous studies have tested for a putative interaction between response cueing and conflict resolution in different ways, mainly using conflict tasks other than flanker tasks (e.g., Simon tasks: Proctor et al. 1992; Wascher and Wolber 2004). Findings from Simon effect tasks have shown that valid response preparation can enlarge rather than reduce the Simon effect (e.g., Wascher and Wolber 2004), suggesting that response preparation effects on conflict processing may be masked by additional stimulus-induced factors such as attention shifts.

A study by Wühr and Heuer (2017) aimed to shed more light on the counterintuitive findings regarding response cueing and the Simon effect. This study most closely resembles the current study design, since it employed a non-spatial response cueing and flanker conflict paradigm in healthy participants and manipulated the frequency of valid and invalid cues to vary response expectations. Trials with incongruent and congruent flankers occurred with equal probability. The results showed that the flanker-induced congruency effect was eliminated in a condition with 100% valid cues (i.e., without response uncertainty). However, in a condition with 75% valid cues, the congruency effect was unaffected by valid or invalid response cues. This finding provides the first evidence for an interaction between the two response control processes induced by cue and flanker stimuli that may depend on response expectations. However, since cues were 100% valid, response expectations were never violated in this condition. A follow-up study by Wühr et al. (2018) replicated the finding of decreased flanker interference (conflict) upon 100% valid response cues with a task modulation that ensured attentional processing of the target. Neither of the two studies (Wühr et al. 2018; Wühr and Heuer 2017), however, tested the effect of expectations regarding conflict occurrence since incongruent trials occurred with the same frequency as congruent trials throughout the task.

The current study aimed to elucidate concurrent expectation-dependent response reprogramming and conflict resolution in a novel response cueing and conflict task. Specifically, the task combined valid and invalid motor response cues with congruent and incongruent target flankers, resulting in four trial types. The invalid-incongruent trial type combined reprogramming of the response after an invalid cue and resolving conflict from flanker interference. Furthermore, varying proportions of these trial types were presented in different blocks to manipulate response and conflict expectations. Based on previous work, we hypothesized to find validity and congruency effects that would be modulated by block-specific expectations. Notably, we asked whether response reprogramming and flanker-induced conflict interacted and further aimed to contextualize this interaction of online response control mechanisms with anticipatory control. This allowed us to assess multiple subprocesses of response control within the same task and to test for their potential non-additivity, comparable to the additive factors method (Sternberg 1969; see also studies on the ANT for a similar procedure, e.g., Fan et al. 2002). While the exact conclusions drawn from interactive effects in the additive factors method are debated (e.g., Pieters 1983; Stafford and Gurney 2011), we assumed that a disproportionate performance decline under simultaneous demands (reprogramming and conflict resolution) - potentially amplified in case of strongly violated expectations - would provide a demonstration of a mutual influence of the different motor-cognitive control subprocesses in this task. This influence may be caused by a partial overlap between the control mechanisms or increased coordinatory demands.

## Methods

### Participants

Thirty-six young healthy participants were recruited for the study. Per inclusion criteria, participants were between the ages of 18–40, native speakers of German, unaffected by any neurological or psychiatric condition, right-handed (handedness was verified with the Edinburgh Handedness Inventory (EHI; mean (± SD) laterality quotient: 78.77 ± 14.64, ranging from 50 to 100; Oldfield 1971), and not taking centrally-acting medication or drugs. All participants gave their written informed consent. The German Psychological Society’s ethics committee approved the study performed following the Ethic’s Code of the World Medical Association (Declaration of Helsinki).

Two participants were excluded after data collection due to exceedingly low accuracy scores (5.83% and 14.09%), suggesting they did not follow the instructions. The performance of three additional participants deviated more than two standard deviations from mean accuracy (91.37 ± 5.51% mean accuracy: lower accuracy limit at 80.34%), leading to their exclusion from further evaluations. Accordingly, data from 31 participants (mean age: 25.13 ± 5.27 years, ranging from 19 to 36 years; 13 female and 18 male) were considered for the analyses. The sample size was based on the behavioral study by Wühr and Heuer (2017), which employed a comparable response cueing/conflict task.

The Barratt Impulsiveness Scale (BIS-11; Patton et al. 1995) was assessed at the beginning of an experimental session and considered for the potential contribution of impulsiveness to individual differences in task performance (Harrison et al. 2009; Landau et al. 2012).

### Experimental set-up

The task was presented on a 47.39 cm x 29.62 cm computer screen while participants sat 94 cm away on a comfortable chair. Left and right index fingers were resting on adjacent response pad keys (response pad “RB-830” by cedrus^®^). Participants positioned their heads on a chin rest during the experiment, individually adjusted to a suitable height. A camera monitored the participants inside the experiment booth to capture any behavior that might have affected task performance.

### Eye-tracking

An EyeLink^®^ 1000 eye-tracker was used to record eye movements during the experiment (sampling rate: 1000 Hz) to control for central fixation. Either left eye or right eye movements were recorded. The eye-tracker was calibrated before the training and before the main task. Participants were instructed to fixate on the screen’s center during a trial. Eye-tracking data that were distorted by glasses/contact lenses or movement (data of nine participants in total) were considered unreliable and thus not included in the analysis.

### Task

The paradigm was based on the motor version of a Posner-type cueing task (Posner 1980; Rushworth et al. 1997) and the arrow version of the Erikson flanker task (Eriksen and Eriksen 1974). Combining these two tasks allowed us to assess motor preparation and reprogramming, response conflict, and their modulation by expectations. The paradigm was programmed with the Presentation^®^ software (Version 18.0, Neurobehavioral Systems, Inc., Berkeley, CA).

### Trial structure

**Fig. 1:**
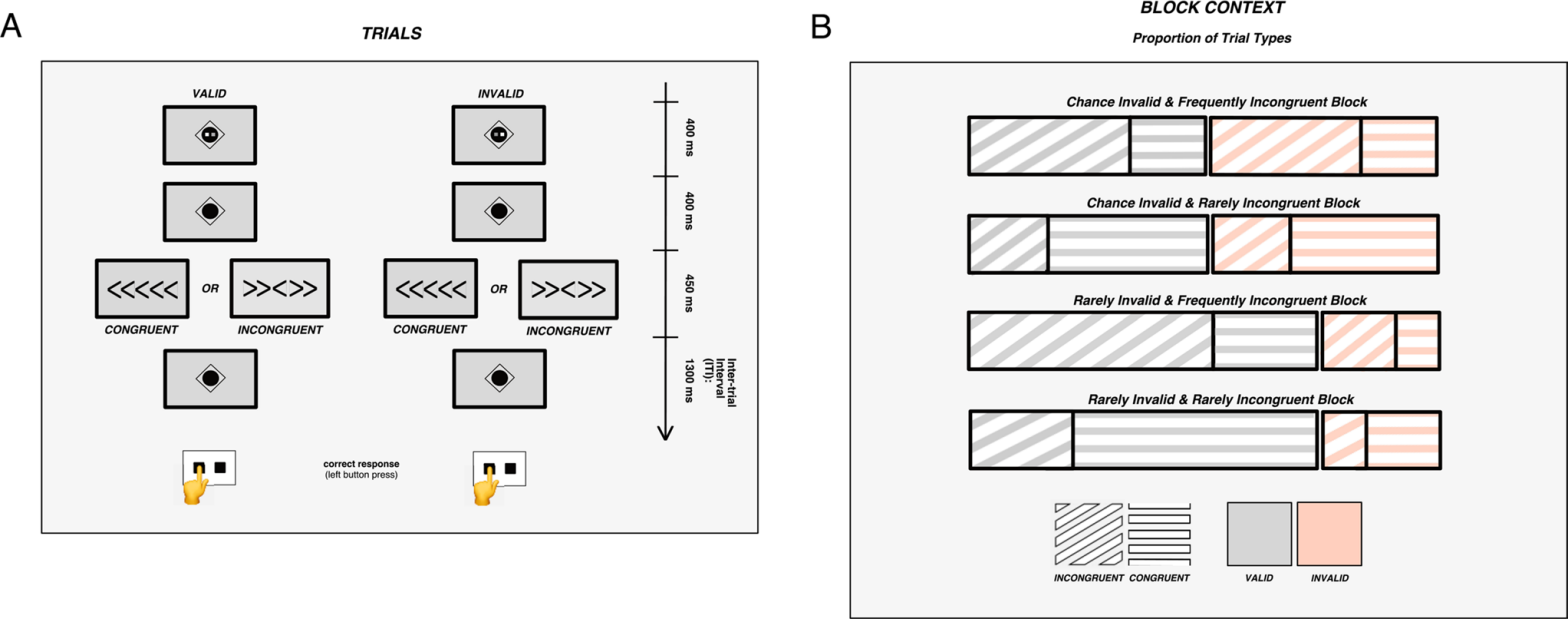
Schematic illustration of the response cueing/conflict paradigm. (**A**) *Trials*: The motor response cues are displayed in the first row. The white square indicated the probable response to the upcoming target (button press with left/right index finger). The target (third row) was shown after a fixation interval (second row). A left/right central target arrow required a left/right-hand button press (left-hand press demanded in this example). In valid trials, the target required the cued response. In invalid trials, the target required the alternative response, i.e., response reprogramming. The central target arrows were presented with either congruent or incongruent peripheral flanker arrows (**B**) *Block context*: Four block types were realized by manipulating the proportions of valid to invalid and congruent to incongruent trials. Each block type was presented thrice in a fixed pseudorandomized order

At the start of each trial, a motor response cue was presented for 400 ms (Fig. 1A; cue dimension: 2 degrees visual angle). The cue depicted the two response keys (left and right square), of which the white key (square) denoted the upcoming response side. In valid trials, the cue indicated the response to the target correctly, while it displayed the incorrect (alternative) response in invalid trials. After the cue, a fixation point (“diamond”) appeared for 400 ms, followed by the target, displayed for 450 ms. The target was a chevron-type arrow in the center of the screen, pointing either to the left or right, thereby demanding a response with the left or right index finger. Two peripheral distractor arrows were displayed on each side of the central target arrow (dimension of one arrow: 1.25 degrees; total stimulus length: 6.61 degrees; arrow spacing: 0.09 degrees). The distractor arrows pointed uniformly either in the same direction as the target (congruent trial) or in the opposite direction (incongruent trial). Following the target presentation, the fixation point reappeared in the inter-trial interval that lasted for 1300 ms, during which responses were still registered. In total, a trial lasted for 2550 ms. Each block contained eight null trials, which showed only the fixation point. These trials lasted for 1700 ms and were used to jitter the inter-trial interval.

### Block structure

#### Main experiment

Varying proportions of valid to invalid and congruent to incongruent trials were employed in different blocks to manipulate expectations regarding the upcoming trial type (Fig. 1B). Each block was presented thrice in a pseudorandom order, which was identical for all participants. In addition, the trial sequence within each block was pseudorandom and identical for all participants (but different for the different block repetitions). This is a standard procedure in studies using computational modeling of stimulus probabilities or contingencies to ensure comparable learning processes (e.g., Iglesias et al. 2013), which we considered as an additional analysis option for this study. Blocks of the same type did not follow each other consecutively. Please refer to Supplementary Figure SF5 for a schematic display of the block sequence.

Regarding validity proportions, valid and invalid trials occurred at chance level (50% valid trials, i.e., validity proportion) in half of the blocks. In these blocks, there was maximal uncertainty about the required motor response. In contrast, in the remaining blocks, most trials were valid (77% validity proportion), thereby reducing the uncertainty about the motor response to the target. This manipulation was similar to previous studies in which the validity proportion varied between 50% and 90% (e.g., Kuhns et al. 2017), allowing for a comparison of conditions with different levels of uncertainty about the upcoming motor response.

Concerning congruency proportions, congruent trials were more likely than incongruent trials (70% congruent trials, i.e., congruency proportion) in half of the blocks, while this relation was reversed in the other half of blocks (30% congruency proportion). These congruency proportions were similar to those realized by Derosiere et al. (2018) and Duque et al. (2016).

Combining the different validity and congruency proportions systematically resulted in four unique block types (Fig. 1B). Each block type was repeated thrice, resulting in 12 blocks of 70 trials each (approximate overall duration: 35 min). Self-paced breaks separated the blocks of the main experiment. The four unique trial types (valid/invalid cue and congruent/incongruent target) within the four unique block types generated a total of 16 distinct conditions, thereby amounting to a 2 (*validity*: valid/invalid) x 2 (*congruency*: congruent/incongruent) x 2 (*validity proportion*: rarely invalid [77% validity proportion]/chance invalid [50% validity proportion]) x 2 (*congruency proportion*: rarely incongruent [70% congruency proportion]/frequently incongruent [30% congruency proportion]) factorial design. Within each condition, left- and right-hand responses were distributed evenly.

Before the start of each block, participants were presented with information on screen as to whether the upcoming block would contain mainly incongruent or congruent trials (to adhere to the Derosiere et al. (2018) and Duque et al. (2016) implementation of congruency proportions). No information about the validity proportion was provided (to follow the Kuhns et al. (2017) approach to implementing validity proportions).

#### Training

Before the main experiment, participants performed a training session with three blocks of 40 trials each (approximately 5 min overall), separated by self-paced breaks between blocks. The first two blocks of the training showed only the central arrow without distractor arrows to accustom the participants to the general speed and sequence of events. The first block comprised mostly valid trials (80% validity proportion), while the second block used an equal proportion of valid and invalid trials (50% validity proportion). The third block introduced the flanking distractor arrows next to the target and mainly contained valid trials (80% validity proportion) and congruent/incongruent trials at chance level (50% congruency proportion). The training trials appeared in a pseudorandomized and fixed order within a block, with no null trials. As in the main task, left- and right-hand responses were evenly distributed within each condition.

### Data analysis

#### Behavioral data

Only trials with correct responses were considered for RT analyses. Anticipatory responses (faster than 100 ms) were discarded. Regarding accuracy scores, missed trials and trials with an incorrect response were treated as erroneous trials. Missed responses amounted on average to 6.40 ± 12.25%.

Our aim was firstly to investigate the main effects of a trial’s validity and congruency as well as their potential interaction, and secondly, whether and how block-wise expectations modulated these trial-level effects. For this, each participant’s condition-specific mean RTs and accuracy scores were analyzed with separate 2 (*validity*: valid/invalid) x 2 (*congruency*: congruent/incongruent) x 2 (*validity proportion*: rarely invalid [77% validity proportion]/chance invalid [50% validity proportion]) x 2 (*congruency proportion*: rarely incongruent [70% congruency proportion]/frequently incongruent [30% congruency proportion]) repeated-measures (RM) ANOVAs. Additionally, we repeated these analyses with each participant’s inverse efficiency (RTs divided by accuracy) to account for differential accuracies between conditions with a compound measure (Snodgrass et al. 1985) (see Supplementary Material). Given the tendency of accuracy results to display skewness, we additionally used the non-parametric Wilcoxon signed-rank test to compare the congruency effect (accuracy incongruent trials minus congruent trials) between conditions, further supporting our accuracy findings.

We expected to find significant main effects of validity and congruency, reflecting the well-described behavioral costs after invalid cues and incongruent flankers. Moreover, we expected significant *validity* x *validity proportion* and *congruency* x *congruency proportion* interactions, reflecting the expectation-dependent modulation of response reprogramming and conflict resolution reported previously (e.g., Kuhns et al. 2017; Duque et al. 2016). We then checked for an interaction effect reflecting an interplay between the respective response control processes in dependency of expectations. We confirmed in an additional analysis that our results of interest would not change when considering only RTs within 3 standard deviations of the individual mean RT (i.e. when discarding trials with outlier RT) (cf. Supplementary Table ST2; the only deviation was a significant two-way interaction of validity proportion and congruency proportion, which, however, was not relevant for our hypotheses, which focused on interaction effects between these block-level and the trial-level factors).

A potential influence of impulsivity on the outcome parameters, assessed with the Barratt Impulsiveness Scale (BIS-11), was tested with additional RM-ANOVAs that included the BIS-11 score as a covariate. The total score and the motor impulsivity sub-score were evaluated in this way to test for associations with individual task performance.

Analyses were carried out with MATLAB (The MathWorks Inc. (2022). MATLAB version: 9.13.0 (R2022a), Natick, Massachusetts: The MathWorks Inc. https://www.mathworks.com) and JASP (JASP Team (2023). JASP (Version 0.17.1)).

### Eye-tracking data

We conducted an eye-tracking analysis to confirm that participants fixated in the center of the screen and refrained from eye movements in the direction of the motor response cues. Pupil coordinates of the right or left eye were analyzed throughout the trial period from cue onset until the end of the target presentation to ensure central fixation. A circle with a diameter of twice the cue dimension (2 degrees to each side) was defined as a region of interest for valid central fixation during this period. Cumulative valid fixation points were expressed as a percentage for each trial and averaged across trials for each participant, of which a grand average over all participants was determined.

The eye-tracking data was analyzed with MATLAB (The MathWorks Inc. (2022). MATLAB version: 9.13.0 (R2022a), Natick, Massachusetts: The MathWorks Inc. https://www.mathworks.com).

## Results

### Eye-tracking data

The analysis of eye-tracking coordinates confirmed a central fixation throughout the cue-target period within a region-of-interest of 2° to each side 92.33 ± 6.15% (ranging from 76.70 to 99.88%) of the time as an average over all participants with valid eye-tracking data (only data without distortion by movements or glasses/contact lenses: 22 out of 31 participants). Hence, participants attended sufficiently to the region-of-interest during a trial.

### Reaction time

#### Validity and congruency effects at trial-level

**Fig. 2:**
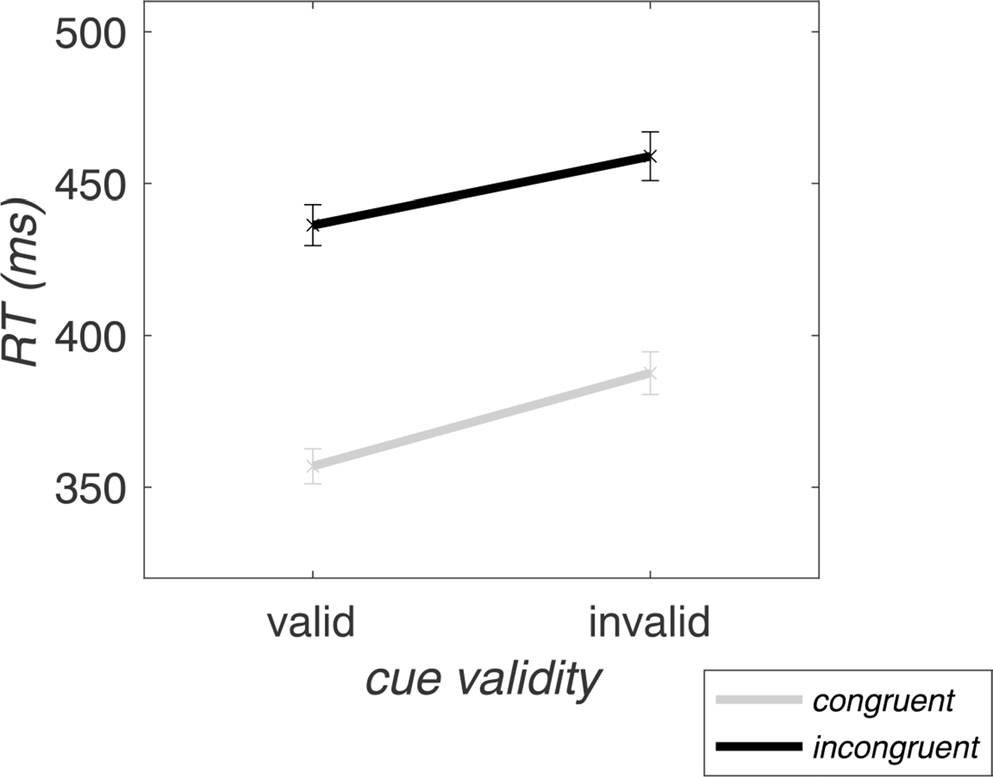
Trial-wise RTs averaged over all block types. The congruency effect is visible as the RT difference (distance) between congruent (gray) and incongruent (black) lines. On the x-axis, the validity effect is reflected in the slope of the lines between valid and invalid trial RTs. The error bars depict the standard error of the mean

The RM-ANOVA on RTs revealed significant main effects of *validity* (*F*_(1,30)_ = 37.68, *p* < .001, η_p_^2^ = 0.56) and *congruency* (*F*_(1,30)_ = 737.88, *p* < .001, η_p_^2^ = 0.96). Invalid cues lead to increased RTs (423.27 ± 40.97 ms) as compared to valid cues (396.64 ± 33.93 ms), and incongruent flankers evoked increased RTs (447.66 ± 39.52 ms) in contrast to congruent flankers (372.25 ± 33.09 ms). Furthermore, *validity* and *congruency* interacted significantly at the trial level (*F*_(1,30)_ = 7.51, *p* = .01, η_p_^2^ = 0.20), with larger congruency effects for valid (79.48 ± 16.51 ms) than invalid trials (71.34 ± 18.51 ms). This effect was mainly driven by faster responses to targets with congruent flankers in validly (as opposed to invalidly) cued trials (valid-congruent: 356.90 ± 32.09 ms; valid-incongruent: 436.38 ± 37.53 ms; invalid-congruent: 387.60 ± 39.02 ms; invalid-incongruent: 458.94 ± 44.79 ms) (see Fig. 2).

### Validity and congruency effects at block-level

Considering the impact of block-specific trial proportions (expectations) in the ANOVA revealed that RTs were significantly shaped by validity and congruency proportions. More specifically, the two-way *validity* x *validity proportion* (*F*_(1,30)_ = 21.84, *p* < .001, η_p_^2^ = 0.42) and *congruency* x *congruency proportion* (*F*_(1,30)_ = 82.20, *p* < .001, η_p_^2^ = 0.73) interactions reached significance. As expected, the validity effect increased with a higher validity proportion (when invalid trials were less expected: 33.17 ± 29.14 ms at 77% validity proportion) compared to the chance level (20.09 ± 20.96 ms at 50% validity proportion). Likewise, the congruency effect was enhanced with a higher congruency proportion (when incongruent trials were less expected: 86.76 ± 18.24 ms at 70% congruency proportion), in contrast to a context of frequent incongruent trials (64.06 ± 15.57 ms at 30% congruency proportion). Next to this, the highest-order effects that reached significance were two significant three-way interactions: *validity* x *congruency* x *congruency proportion* (*F*_(1,30)_ = 6.20, *p* < .05, η_p_^2^ = 0.17) as well as *validity* x *congruency* x *validity proportion* (*F*_(1,30)_ = 10.89, *p* < .01, η_p_^2^ = 0.27). These interactions reflected modulations of the validity-by-congruency interaction by expectations about the motor response and conflict occurrence and are described below in more detail. The four-way interaction was not significant. A complete list of effects that resulted from the RM-ANOVA at inter- and intra-trial- and block-levels is provided in Supplementary Table ST1.

### Response expectation-dependent modulation of the validity effect by congruency

**Fig. 3:**
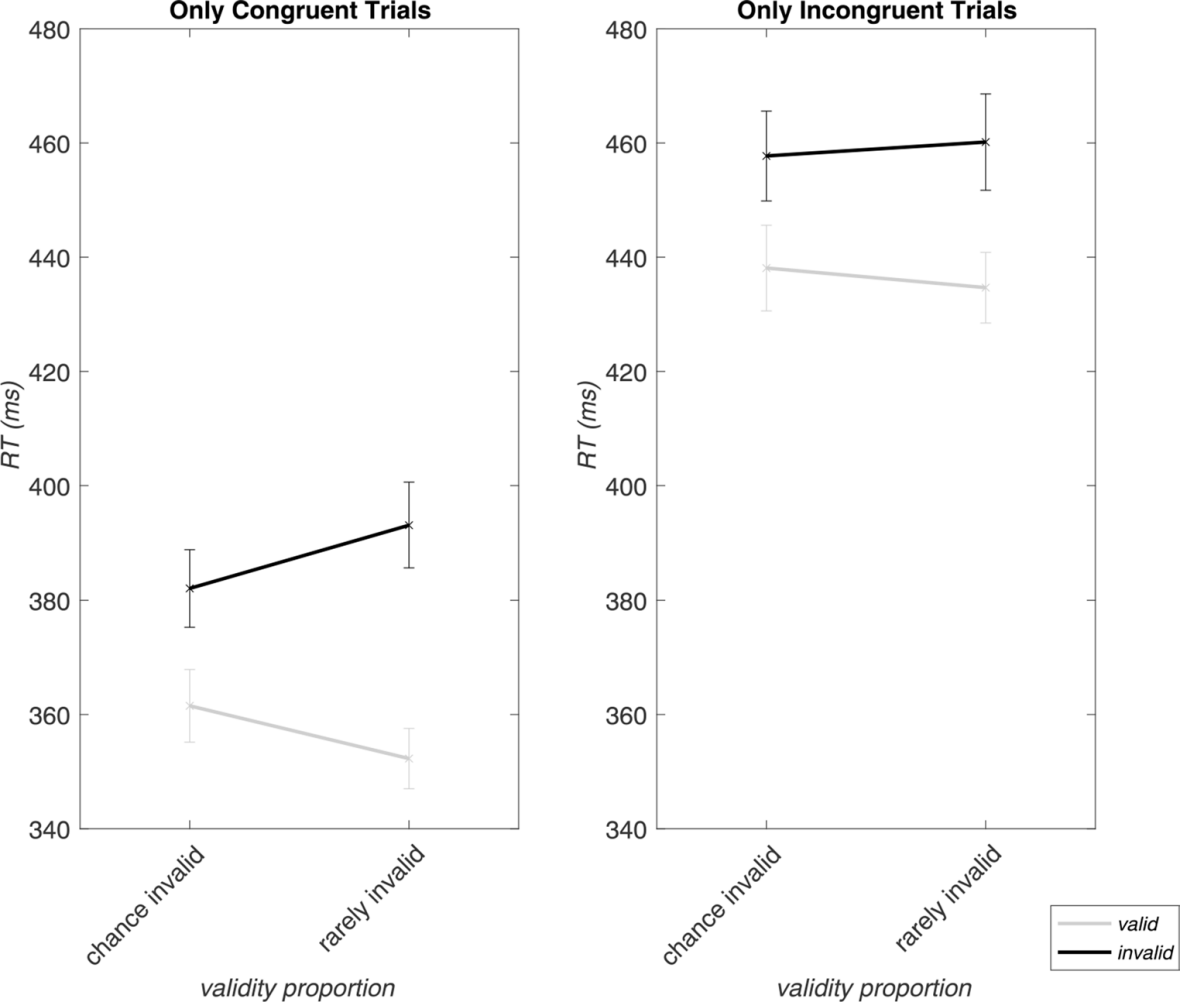
Modulation of the RTs in invalid and valid trials (i.e., the validity effect) by response expectations (validity proportion), separated by trial congruency. This figure visualizes the three-way interaction for RTs of *validity*, *congruency*, and *validity proportion*. Line graphs show differences between chance (50% validity proportion) and rarely invalid (77% validity proportion) block types for valid (grey lines) and invalid (black lines) trials. The error bars show standard errors of the mean

A closer look at the three-way interaction of *validity*, *congruency*, and *validity proportion* revealed that for congruent trials (no flanker-induced conflict), RTs were slower for invalid trials when they were rare (77% validity proportion; 393.14 ± 41.61 ms) than when they occurred at chance level (50% validity proportion; 382.07 ± 37.82 ms). In a complementary manner, valid cueing induced faster RTs (352.28 ± 29.40 ms) within rarely invalid blocks, compared to chance invalid blocks (361.52 ± 35.41 ms) in congruent trials. Therefore, without conflict by incongruent flankers, the RT validity effect (difference between invalid and valid trial RTs) increased as expected when invalid trials were rare. In contrast, in incongruent trials (conflict present), in which RTs were generally higher, the modulation by validity proportion was attenuated (i.e., varying response expectations; see Fig. 3).

### Conflict expectation-dependent modulation of the congruency effect by validity

**Fig. 4:**
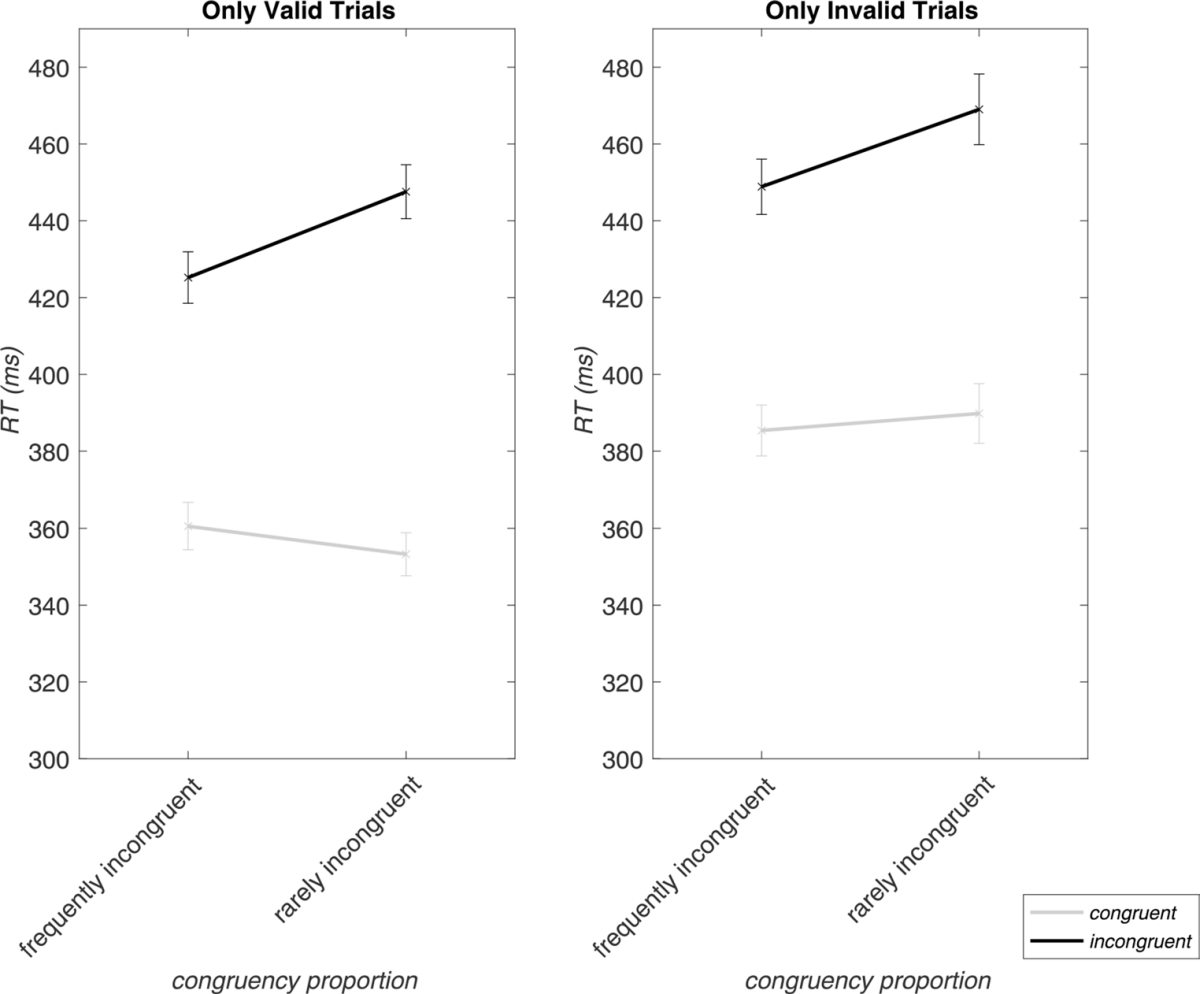
Modulation of the RTs in incongruent and congruent trials (i.e., the congruency effect) by conflict expectations (congruency proportion) for valid and invalid trials. The x-axis depicts frequently incongruent (30% congruency proportion) and rarely incongruent (70% congruency proportion) block types with mean RTs for congruent (grey lines) as well as incongruent (black lines) trials. Error bars display the standard errors of the mean. This figure depicts the three-way interaction for RTs of *validity*, *congruency*, and *congruency proportion*

As indicated by the three-way interaction of *validity*, *congruency*, and *congruency proportion*, the modulation of the congruency effect (RT incongruent minus RT congruent trials) was dependent on the validity of a trial. While the congruency effect was, as expected, generally larger in the context of rare incongruent trials (70% congruency proportion) compared to frequent incongruent trials (30% congruency proportion), the effect was further enhanced when a trial was also valid (i.e., when response expectations were not violated) as compared to invalid (Fig. 4).

**Fig. 5:**
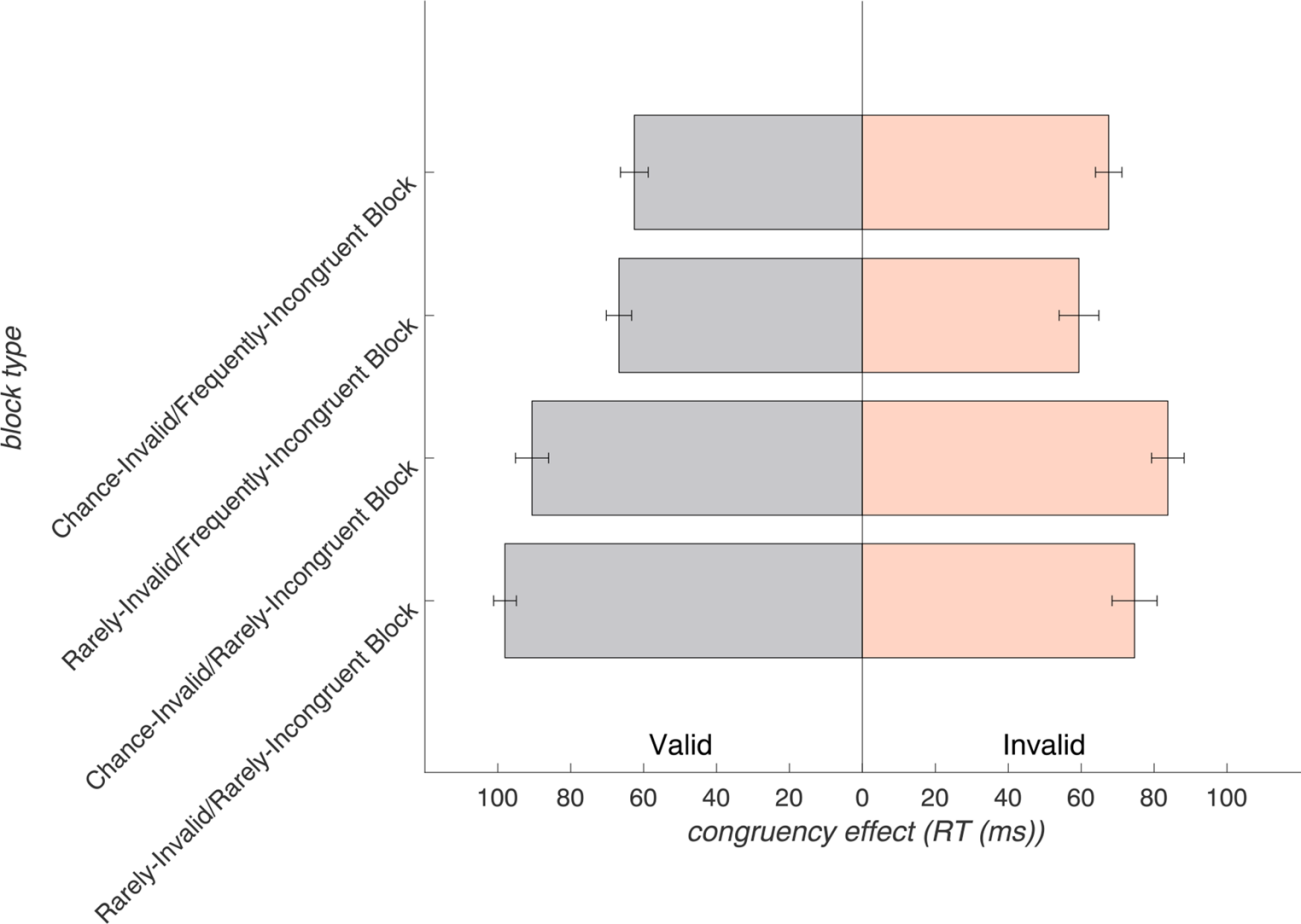
The congruency effect (RT incongruent trials – congruent trials) is shown for each block type (y-axis), separated by valid (gray bars) and invalid (orange bars) trials on the x-axis. For valid trials, the rarely-incongruent blocks clearly show the expected larger congruency effect compared to the frequently-incongruent blocks, which is also larger than in the invalid rarely-incongruent blocks. Error bars depict the standard error of the mean

Figure 5 summarizes the congruency effects in the different blocks. A summary figure and table with data for all conditions are provided in the Supplementary Material (Supplementary Figure SF4 and Table ST3)

### Accuracy

#### Validity and congruency effects at trial-level

**Fig. 6:**
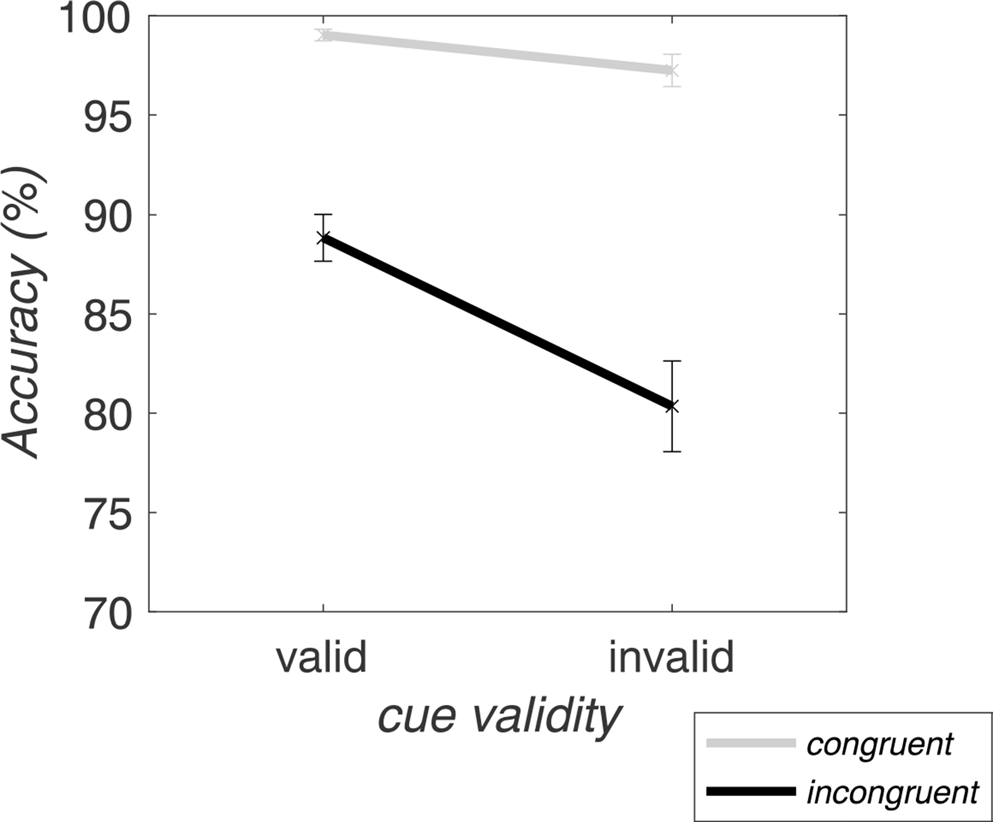
Accuracy on trial-level, averaged over all block types. The difference between the congruent (gray) and incongruent (black) lines shows the congruency effect, which is larger for invalid than valid trials. The validity effect is visible as the slope of the lines between valid and invalid trials (on the x-axis). Error bars indicate the standard error of the mean

As for the RM-ANOVA on RTs, the RM-ANOVA on accuracy yielded significant main effects of *validity* (*F*_(1,30)_ = 21.24, *p* < .001, η_p_^2^ = 0.41) and *congruency* (*F*_(1,30)_ = 108.53, *p* < .001, η_p_^2^ = 0.78), with lower accuracy in invalid (88.80 ± 8.14%) than valid (93.94 ± 3.72%) and in incongruent (84.60 ± 8.84%) than congruent (98.14 ± 2.97%) trials. Again, as for RTs, the trial-level interaction between *validity* and *congruency* was significant (*F*_(1,30)_ = 22.48, *p* < .001, η_p_^2^ = 0.43). However, in contrast to RTs, it manifested in larger congruency effects for invalid (16.90 ± 9.98%) than valid trials (10.19 ± 6.01%) (Fig. 6).

#### Validity and congruency effects at block-level

Block-level effects contributed to a significant four-way interaction in the RM-ANOVA (*F*_(1,30)_ = 7.11, *p* < .05, η_p_^2^ = 0.19). Additionally, every lower-order effect was significant, except for the three-way interaction of *validity* x *congruency* x *validity proportion*. A complete RM-ANOVA table for accuracy effects is presented in Supplementary Table ST1. Repeating this analysis with inverse efficiency scores revealed similar effects (see Supplementary Material).

**Fig. 7:**
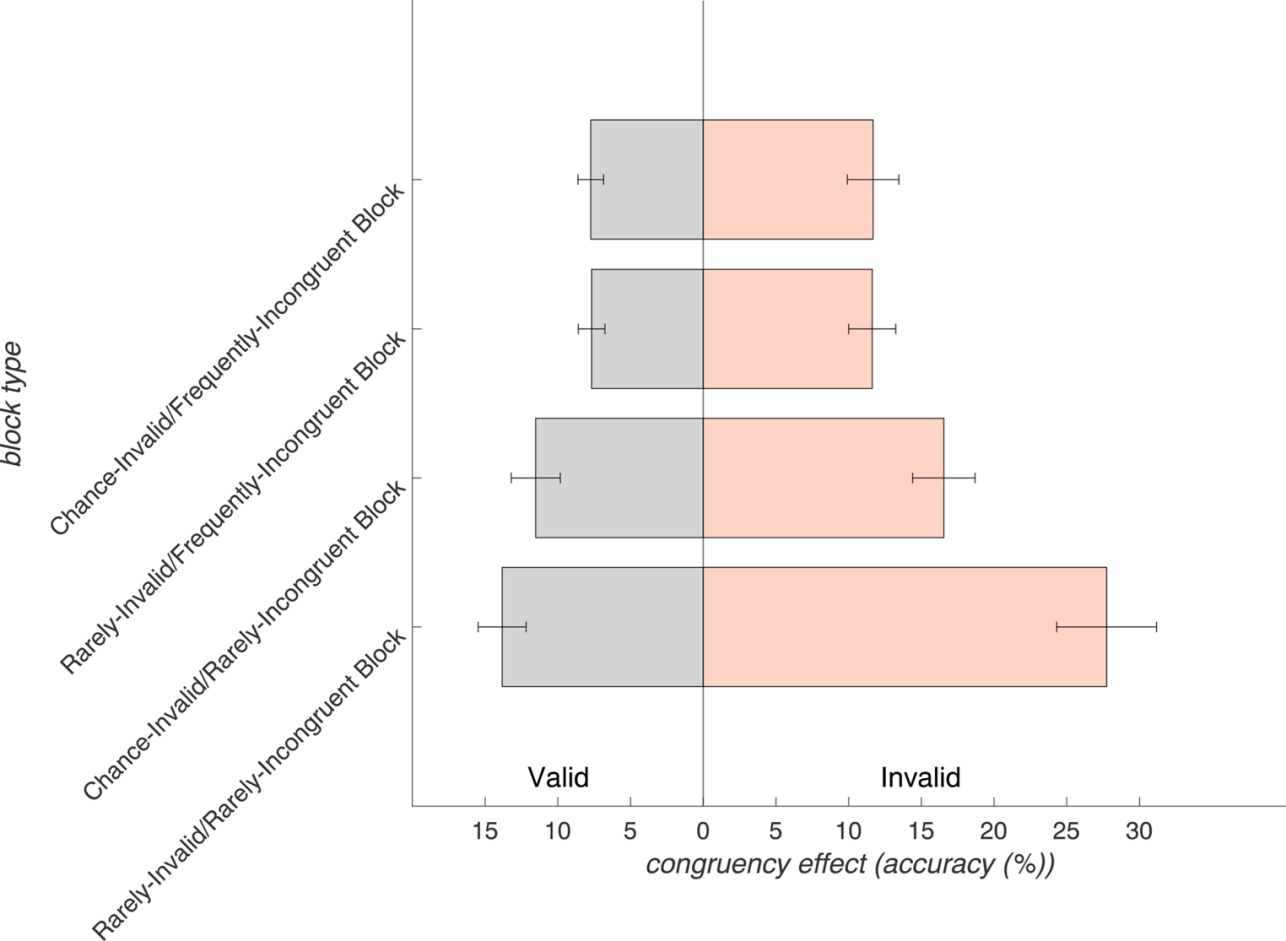
Congruency effects (accuracy incongruent trials – congruent trials) for each block type (y-axis), divided by valid (gray bars) and invalid (orange bars) trials on the x-axis. This visualizes the disproportionate increase of the congruency effect within invalid trials for the rarely-invalid/rarely-incongruent block. The error bars show standard errors of the mean

The significant four-way interaction became most apparent within the incongruent trials, where the distinctively lowest accuracy was observed for invalid-incongruent trials in the context of strongly violated expectations for both response and conflict (rare invalid [77% validity proportion]/rare incongruent [70% congruency proportion] blocks, see Fig. 7). This finding reflects that the trial- and block-specific effects on accuracy were not additive but disproportionally enhanced in this condition (68.28 ± 21.56% accuracy; post-hoc paired samples *t*-tests against all other conditions were significant (all *p* < .001) when applying Bonferroni correction). Additionally, due to the skewness of accuracy measures, we applied non-parametric Wilcoxon signed-rank tests to compare the congruency effect of this condition against those of the remaining seven conditions (Fig. 7). These comparisons revealed significant differences after Bonferroni correction (all *p* < .05), affirming our findings obtained with parametric tests.

### BIS-11 score

The mean (±SD) BIS-11 total score amounted to 57.58 ± 5.99, and the motor impulsivity sub-score to 13.26 ± 2.00. The RM-ANOVAs on RTs, accuracy, and inverse efficiency that included either BIS-11 score as a covariate did not reveal any significant effects (all *p* > .1) of those impulsivity measures on the response control processes assessed in our paradigm.

## Discussion

This study employed a novel response cueing/conflict paradigm that assessed expectation-dependent response reprogramming and conflict resolution, along with their behavioral interactions in RTs and accuracy. Previous studies have employed motor response cues to induce response preparation and reported lower performance for invalid rather than valid cueing conditions. The current study observed this validity effect for both behavioral outcomes. Similarly, peripheral arrows that are presented together with a target stimulus have been shown to decrease performance when incongruent (i.e., associated with an alternative motor response) rather than congruent to the target. Again, we replicated this congruency effect for RTs and accuracy. Moreover, in both measures, our findings revealed significant interactions between validity and congruency effects.

Consistent with related studies (Derosiere et al. 2018; Duque et al. 2016; Kuhns et al. 2017), we observed that the magnitude of the validity effect was dependent on the expectations of invalid cues, while the size of the congruency effect was influenced by expectations regarding incongruent stimuli. These effects were evident in both RTs and accuracy. Consequently, the varying frequencies of trial types in different blocks influenced both online response reprogramming and conflict resolution, potentially through anticipatory control mechanisms. Specifically, as reported in Kuhns et al. (2017), the reprogramming of a prepared response was slower and more often unsuccessful when invalid trials were less frequent and thus less expected. In contrast, stronger motor preparation due to predictive cues facilitated response execution in valid trials. Similarly, less frequent and thus less expected incongruent trials amplified the congruency effect (as in, e.g., Duque et al. 2016).

Our data extend these findings by showing interactions between validity and congruency at the trial-level, and higher-level interactions with expectation-dependent modulations. Validity-by-congruency interactions were present for RT and accuracy, with different conditions driving the interactions. Whereas the congruency effect in accuracy was enhanced via a reduction of accuracy with invalid cueing in incongruent trials, the congruency effect in RTs was enhanced via facilitation by valid cues in congruent trials. This latter effect bears similarity to findings from the Simon effect (e.g., Wascher and Wolber 2004). Hence, although our data show a robust main effect of validity on both RTs and accuracy, preparation processes for a response and for stimuli processing cannot be unequivocally disentangled here, as the cue is indicative of both response type and stimulus appearance. Therefore, it cannot be ruled out that the validity effect in our study reflects additional factors beyond response preparation. Still, our finding aligns with previous studies investigating expectation-dependent response preparation with response cues that have been shown to rely on other mechanisms than feature or spatial cueing (e.g., Kuhns et al. 2017).

Moreover, for RTs and accuracy, the expectation-dependent modulations of validity and congruency effects were interdependent, i.e., the validity effect modulation depended on the congruency of the target display and the congruency effect modulation depended on the validity of the cue. The four-way interaction among all factors was not significant for RTs, but for accuracy. This was driven by a disproportionate (more-than-additive) decrease in performance when a strong expectation about an imminent response was violated, and the target was unexpectedly combined with incongruent flanker stimuli (invalid-incongruent trials in rarely-invalid/rarely-incongruent blocks). Notably, this effect could not merely be attributed to the rare occurrence (i.e., general surprise) of this trial type: Since the task also comprised a block with reversed congruency proportions, the number of trials with rare invalid cues and rare incongruent flankers was identical to the number of trials with rare invalid cues and rare congruent flankers (see Fig. 1B), where this performance decrement was not observed. Instead, the interaction effect presumably resulted from simultaneous demands for online control of response reprogramming and conflict resolution, in particular when unexpected. This may be attributed to partially shared mechanisms of the different online control subprocesses leading to mutual interference, or to a bottleneck in coordinatory processes. While the anticipatory conflict adaptation account of the proportion congruency effect is debated (e.g., Braem et al. 2019), the validity proportion effects more directly reflect cue-induced preparatory response control. Our finding that both manipulations interactively shape accuracy in invalid-incongruent trials may point to similar or converging anticipatory mechanisms. These could be characterized in future neuroimaging or electrophysiological studies with the present task.

Relating these results to other cueing/conflict paradigms in the literature, the combined response cueing/flanker task used by Wühr and Heuer (2017) is most comparable to the present study. In contrast to their results, the present study found significant interactions between validity and congruency despite using probabilistic cues that were valid in 50% or 77% (not 100%) of the trials. Differences in the stimuli and study design may account for these discrepancies. Notably, the study by Wühr and Heuer (2017) used letters as flankers, whereas the present study used arrows, which may elicit more automatic and, thus, more robust response activation (Ridderinkhof et al. 2021), potentially promoting an interaction between the cued response and target congruency in our task. Furthermore, Wühr and Heuer (2017) did not manipulate conflict expectations, and manipulated the validity proportion in different experiments with different groups of participants.

One could speculate that differences between RTs and accuracy in the present study may be due to strategies prioritizing speed over accuracy, resulting in increased errors without the corresponding change in RTs. Along these lines, Draheim et al. (2021) found that the RT measures alone may inadequately represent performance in flanker and Stroop tasks due to individual speed-accuracy trade-offs. The studies by Duque et al. (2016) and Derosiere et al. (2018) employed similar manipulations of block-wise congruency proportions. Analyses regarding the directionality of the interaction effect revealed that the two studies obtained the same pattern of results for accuracy, while their findings differed for RTs. More specifically, the interaction was driven by the difference in RTs in incongruent trials in Duque et al. (2016) and by the RT difference in congruent trials in Derosiere et al. (2018). Concerning accuracy, however, both studies found that the incongruent trials in the rarely incongruent context were more prone to errors.

While studies that manipulate motor preparation and conflict resolution within the same task are scarce, similar interactions between validity and congruency effects have been extensively studied in the attention domain. For instance, the Attention Network Test (ANT; Fan et al. 2002) assesses the functions of three partially independent attention networks: alerting, spatial orienting, and executive attention (conflict resolution of incongruent flankers). In the revised ANT task (ANT-R; Fan et al. 2009), invalid cues were introduced alongside predictive valid cues. The results revealed uncorrelated behavioral scores (Fan et al. 2002) and differential involvement of specific brain networks (Fan, Kolster, Fan et al. 2007a, b) and brain oscillatory patterns (Fan, Byrne, Fan et al. 2007a, b) for each of the attentional subdomains. Nonetheless, the subdomains also seem to draw on partially shared resources. When contrasting orienting (spatial and temporal information) and alerting (temporal information only) cues, it was found that alerting cues increased the congruency effect, while orienting cues interacted positively with the executive domain by reducing the congruency effect when the target location was validly cued (Fan et al. 2009) and modulated congruency-related activation in the insula (Trautwein et al. 2016). At the neural level, the anterior cingulate cortex (ACC), a crucial structure for conflict processing (Matsumoto and Tanaka 2004; Pochon et al. 2008), may also be involved in uncertainty processing (Behrens et al. 2007) of cues containing alerting (i.e., no spatial) information, thereby increasing the processing load. This relates to the potential underlying neural processes associated with our behavioral effects, as the ACC might be activated by uncertainty during response reprogramming after violated expectations and by conflict processing required by an incongruent target (the invalid-incongruent condition in our task). Supporting this, Kuhns et al. (2017) reported increased ACC (and left parietal) activation in an fMRI study when invalid motor cues were more unexpected. In the current motor response cueing/conflict task, a shared reliance on specific cingulo-opercular and fronto-parietal structures may potentially account for the pronounced decrease in performance observed in our rare invalid-incongruent condition. Moreover, neurons in the primary motor cortex, onto which all modulatory signals converge, could also contribute to a shared reliance.

Future studies could employ the present task to test whether shared network activations, putatively involving the ACC, underlie the behavioral interactions of unexpected response reprogramming and conflict resolution reported in this study. In a clinical setting, this task could be employed in patients with impaired cortico-basal ganglia-thalamo-cortical (CBGTC) pathways to provide a more profound understanding of the common structure behind motor-cognitive response control mechanisms and their neural underpinnings.

## Conclusion

The current study explored the interplay between anticipatory and online response control mechanisms, revealing a behavioral interaction between expectation-dependent response reprogramming and conflict resolution. These results suggest coordinated and partially overlapping anticipatory and online response control mechanisms within motor-cognitive networks.

## Electronic supplementary material

Below is the link to the electronic supplementary material.

Supplementary Material 1

## Data Availability

The behavioral and eye-tracking data files and the relevant code used for analyses (MATLAB) is available on the online GIN repository G-Node (https://gin.g-node.org/asauter/2023_Sauter_ResponseControl).
