# Supplementary material for: Response and conflict expectations shape motor responses interactively
Source: Exp Brain Res. 2024 Sep 24;242(11):2599–612. doi: 10.1007/s00221-024-06920-w (PMC11527934; doi:10.1007/s00221-024-06920-w)

**Supplementary Information**

***Complementary Analysis of Inverse Efficiency***

Considering the differential RT and accuracy patterns, we analyzed the inverse efficiency score (RT/accuracy) similarly, to assess combined performance. Overall, the results of this analysis mirror the accuracy effects.

*Validity and Congruency Effects at Trial-Level*

As for RT and accuracy alone, the analysis of inverse efficiency as a compound measure of both read-outs revealed significant main effects of *validity* (*F*_(1,30)_ = 25.26, *p* < .001, η_p_^2^ = 0.46) and *congruency* (*F*_(1,30)_ = 157.13, *p* < .001, η_p_^2^ = 0.84). Inverse efficiency scores were higher for invalid (506.41 ± 86.49 ms) in comparison to valid trials (427.55 ± 32.16 ms), as well as for incongruent (553.58 ± 79.87 ms) as compared to congruent (380.38 ± 35.75 ms) trials. Moreover, as in the separate analyses of RTs and accuracy, a significant two-way interaction between trial-wise *validity* and *congruency* was observed (*F*_(1,30)_ = 12.37, *p* = .001, η_p_^2^ = 0.29). For the inverse efficiency, larger congruency effects were observed in invalid (212.28 ± 135.41 ms) compared to valid trials (134.12 ± 33.97 ms), which points in the same direction as the accuracy analysis. Trial-level effects are visualized in Supplementary Figure SF1.

**Supplementary Fig. SF1** Visualization of the inverse efficiency at the trial level, averaged over all block types. The congruency effect becomes visible as the difference between congruent (gray) and incongruent (black) lines, showing a more substantial increase in invalid compared to valid trials (depicted on the x-axis). Error bars display the standard error of the mean

*Validity and Congruency Effects at Block-Level*

The RM-ANOVA for inverse efficiency scores yielded a significant four-way interaction (*F*_(1,30)_ = 9.17, *p* < .01, η_p_^2^ = 0.23; Supplementary Figure SF4C), likewise to the accuracy analysis. Every lower-order effect was also significant (please refer to Supplementary Table ST1 for a complete list of the RM-ANOVA outcome).

With focus on incongruent trials, inverse efficiency scores mirrored the accuracy scores (Supplementary Figure SF4C) with the rare invalid (77% validity proportion)/rare incongruent (70% congruency proportion) trials presenting as the condition with disproportionally decreased performance compared to every other condition (as shown in slowest inverse efficiency scores: 788.16 ± 350.29 ms; every post-hoc paired samples *t*-test comparison with this condition was significant (all *p* < .001) after Bonferroni correction). This performance decrement is displayed again in Supplementary Figure SF2 regarding the congruency effect (inverse efficiency incongruent - congruent trials) that disproportionally increased for invalid trials within the rarely-invalid/rarely-incongruent block

**Supplementary Fig. SF2** Disproportionate increase of the congruency effect (inverse efficiency incongruent trials – congruent trials) for invalid trials within the rarely-invalid/rarely-incongruent block. The x-axis divides trial types between valid (grey bars) and invalid (orange bars). In addition, the y-axis shows the four different block types. Taken together, this graph visualizes how the congruency effect depends on block type and trial validity. The error bars indicate the standard errors of the mean

**Supplementary Fig. SF3** Display of the RT congruency effect (RT incongruent – RT congruent trials) with respect to validity proportion in only valid (left side) or only invalid trials (right side). On the x-axis, the difference between chance invalid (50% validity proportion) and rarely invalid block types is shown. Congruent trials are shown in gray, and incongruent trials in black. When a primarily reliable response cue, as in the rarely invalid blocks, violated expectations (only invalid trials), the facilitation effect of congruent trials decreases compared to the chance-level response cue in chance invalid blocks. The opposite is visible in only valid trials: congruent trials benefit more from the flanker facilitation effect when response expectations with mostly reliable cues are not violated, compared to chance response cues

**Supplementary Fig. SF4** Modulation of validity and congruency effects by response and conflict expectations. Line graphs show all sixteen conditions for each DV separately: (A) RTs, (B) accuracy, and (C) inverse efficiency (RT divided by accuracy). For (A), (B), (C): Incongruent trials are depicted in pink shades, while congruent trials are green-shaded. The x-axis is divided into valid and invalid trial types. Furthermore, the validity proportion is indicated by darker (rarely invalid block type: 77% validity proportion) and lighter (chance invalid block type: 50% validity proportion) color shadings. In addition, the congruency proportion of the respective block types is indicated by the line type: dotted lines represent frequently incongruent blocks (30% congruency proportion), while continuous lines represent rarely incongruent blocks (70% congruency proportion). Error bars depict the standard errors of the mean

**Supplementary Fig. SF5** Visualization of the response cueing/conflict task structure showing the consecutive order of blocks. Each of the four unique block types (depicted in different colors and patterns) occurred thrice during the task, each time with a unique pseudorandom trial sequence (the same across participants). Each block contained 70 trials, of which 62 were regular and 8 were null trials. In total, the task consisted of 840 trials (12 blocks x 70 trials), of which 96 trials were null trials. Self-paced breaks separated the blocks. The task lasted approximately 35 minutes in total

**Supplementary Table ST1** Overview of RM-ANOVA results for RTs, accuracy, and inverse efficiency (RT divided by accuracy). Presented are *F* and *p* values and the partial eta squared for all main effects, two-way, three-way, and four-way interactions

**
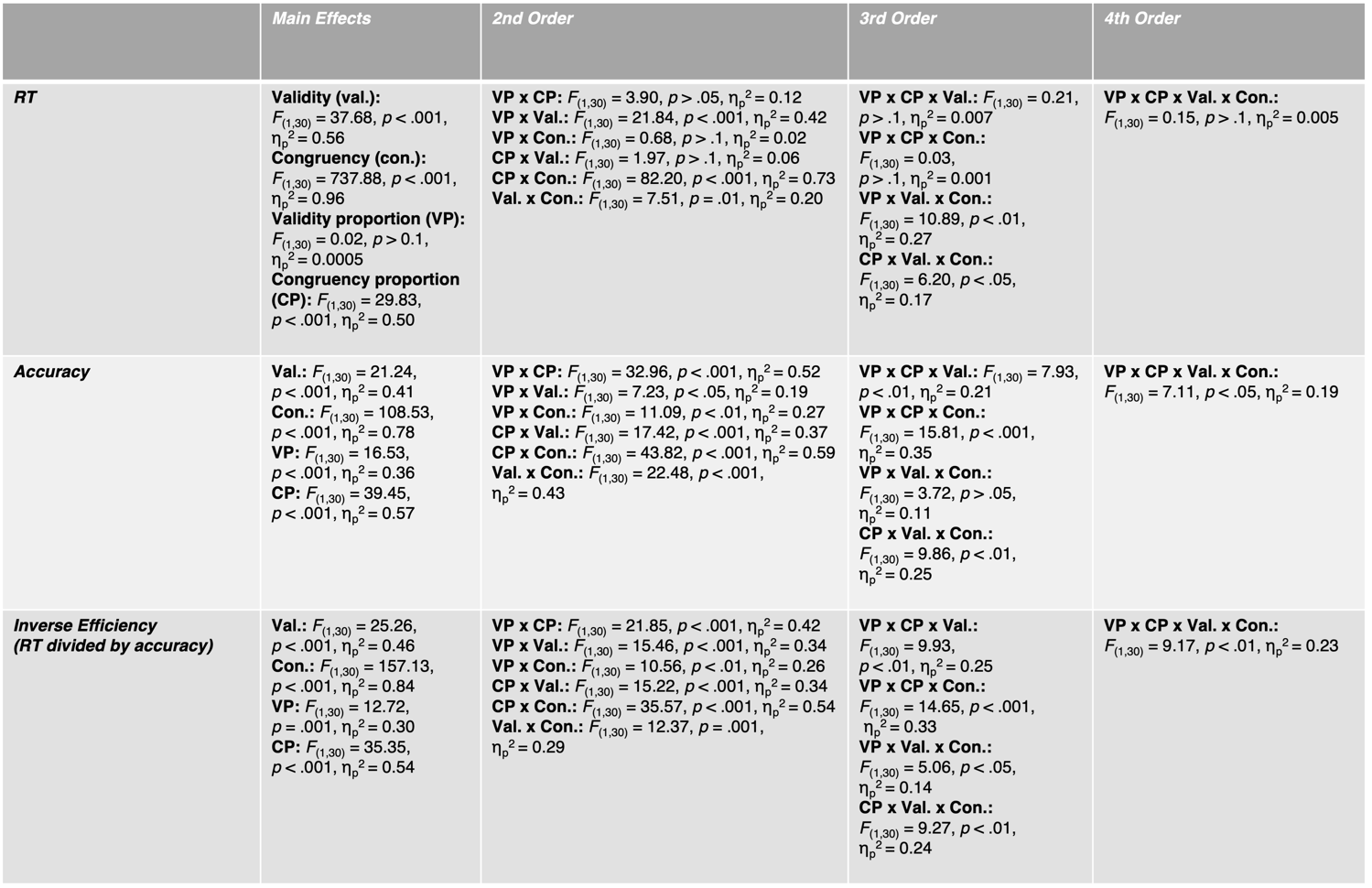
**

**Supplementary Table ST2** Display of the RT RM-ANOVA results considering only RTs within 3 standard deviations from the individual mean RT. Presented are *F* and *p* values and the partial eta squared for all main effects, two-way, three-way, and four-way interactions

**
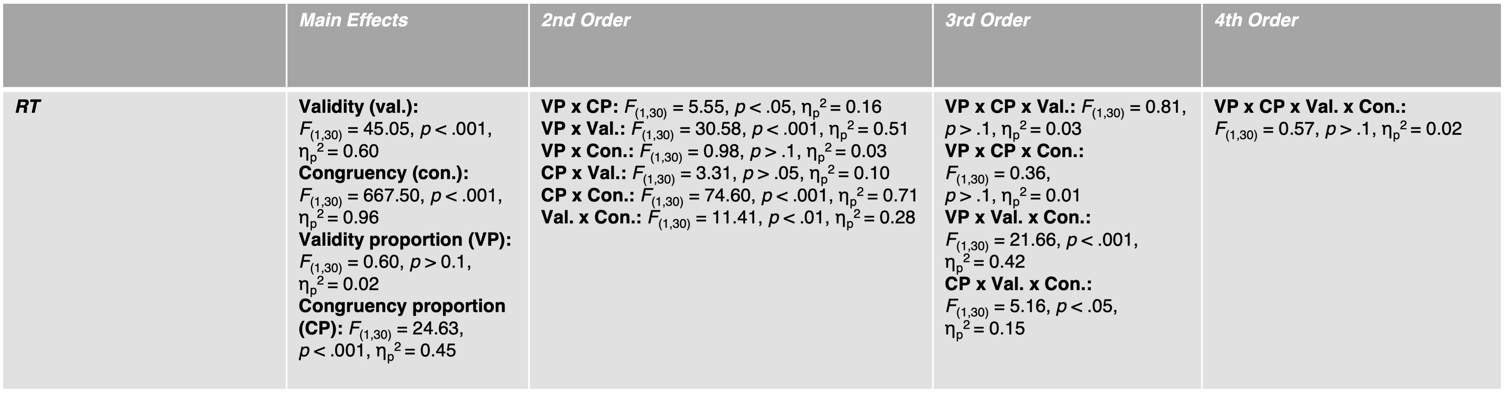
**

**Supplementary Table ST3** Mean (+ SD) values per condition, averaged over participants, for RTs, accuracy, and inverse efficiency (RT divided by accuracy). Block types are specified in the second column, and trial types are depicted in the first row


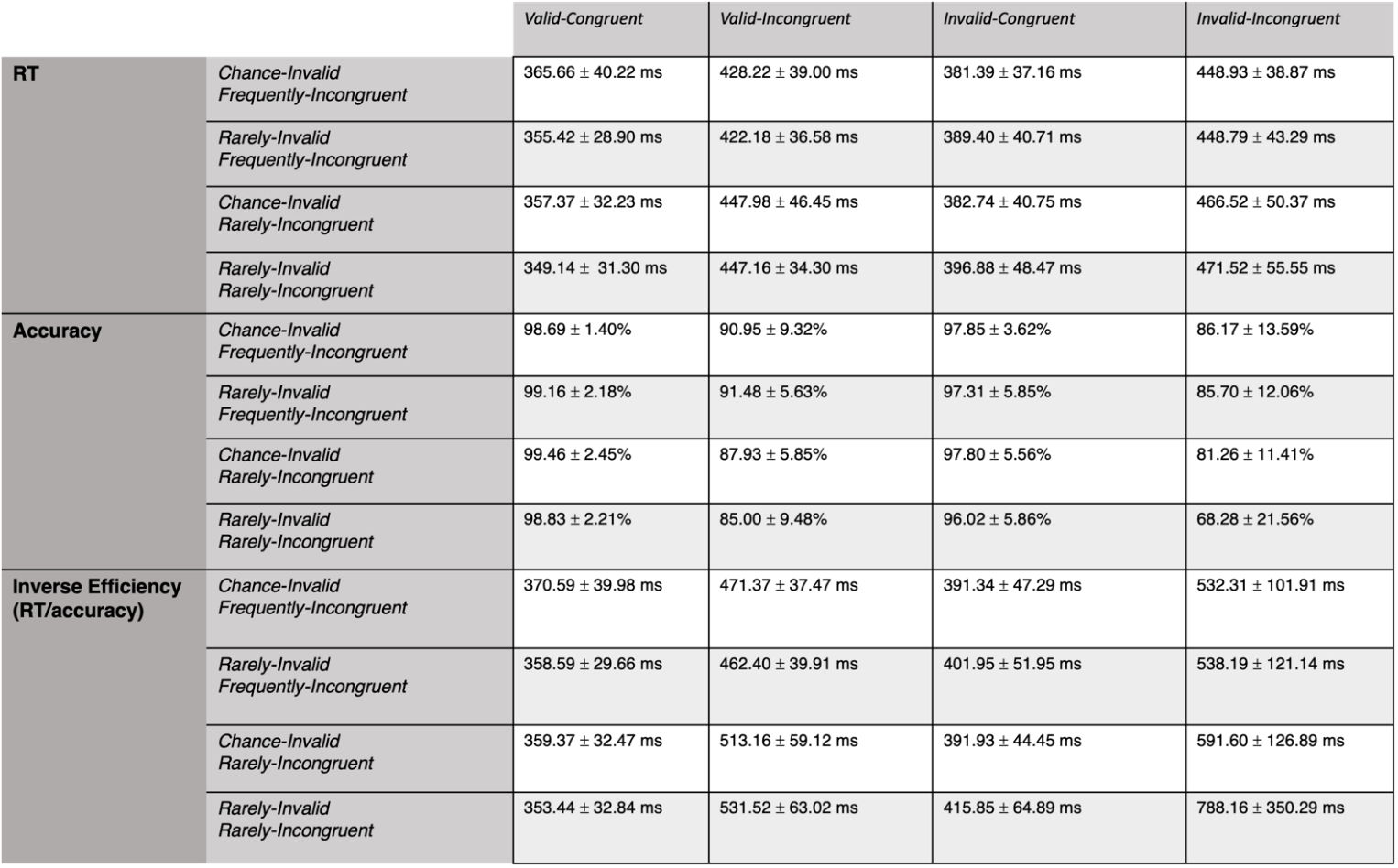

Supplement: Supplementary file 1 — Supplementary Material 1 [file 221_2024_6920_MOESM1_ESM.docx]
